# Risk Factors of Requiring Tracheostomy in COVID-19 Patients: A Retrospective Analysis of Intubated Patients

**DOI:** 10.3390/jcm15041342

**Published:** 2026-02-08

**Authors:** Annika Bharwani, Laith A. Ayasa, Camilo A. Avendano, Raymond C. Parrish, Juan C. Lara, Juan C. Cedeno, Kai Swenson, Jason Beattie, Adnan Majid, Mihir S. Parikh

**Affiliations:** 1 Division of Thoracic Surgery and Interventional Pulmonology, Department of Surgery, Beth Israel Deaconess Medical Center, Harvard Medical School, Boston, MA 02215, USA; abharwan@bidmc.harvard.edu (A.B.); juankcserna@gmail.com (J.C.C.); kswenso1@bidmc.harvard.edu (K.S.); jbeattie@bidmc.harvard.edu (J.B.); amajid@bidmc.harvard.edu (A.M.); 2 General Internal Medicine, Jackson Memorial Hospital, University of Miami Miller School of Medicine, Miami, FL 33136, USA; 3 General Internal Medicine, Montefiore Medical Center, Albert Einstein College of Medicine, Bronx, NY 10461, USA

**Keywords:** COVID-19, tracheostomy, critical care

## Abstract

**Background**: Prolonged mechanical ventilation and tracheostomy in patients with COVID-19 is associated with longer hospital stays. Guidance on which patients are at risk for tracheostomy due to the progression of COVID-19 is limited. **Objectives**: This study aimed to identify risk factors associated with the need for tracheostomy in patients intubated for COVID-19 between 1 March and 31 December 2020. **Methods**: The methodology for this study involved a single-center retrospective analysis of 120 patients who were intubated due to COVID-19 infection between 1 March 2020 and 31 December 2020. A comparison of variables was performed using the Wilcoxon test, Chi-squared test, and Fisher’s exact test alongside univariate analysis. **Results**: Several risk factors were found to be significantly associated with the need for tracheostomy, including age, P/F ratio, creatinine level, and history of arrhythmia. **Conclusions**: Initial exploration indicates the presence of certain factors that can help us understand future need for tracheostomy earlier in the patient’s clinical course. Further analysis should be performed with a larger sample size to validate these findings and increase the generalizability of the present study.

## 1. Introduction

COVID-19 infection cases were first reported in the United States in early 2020 [1]. Soon afterwards, hospitals across the United States were inundated with large numbers of patients suffering from hypoxemic respiratory failure secondary to COVID-19 infection, with approximately 5–15% of hospitalized patients ultimately needing to be intubated [2,3]. Previously, we have presented findings demonstrating that prolonged mechanical ventilation in the treatment of COVID-19 is associated with a higher rate of complications [4]. Globally, up to one in five critically ill patients with COVID-19 infection required invasive mechanical ventilation, with mortality rates exceeding 40% among ventilated cohorts [5,6]. This surge placed immense strain on ICU capacity, ventilator availability, and staffing resources.

During the early stages of the pandemic, the criteria qualifying patients for intubation evolved constantly as clinicians aimed to balance hypoxemia management with avoidance of emergent airway interventions [7]. Concerns regarding aerosol generation and healthcare worker safety have further impacted airway management strategies, often leading to delayed tracheostomy or a preference for specific procedural approaches [8]. In addition, institutional practices varied with regards to surgical versus percutaneous tracheostomy techniques, which lead to even more heterogeneity in timing and patient selection [9].

Tracheostomy has rapidly become an essential intervention for prolonged mechanical ventilation, secretion management, and facilitation of weaning. It is worth noting, however, that this procedure carries unique uncertainty. Early anecdotal reports led to wide variability in timing and indications; moreover, guidance on when to perform tracheostomies and which patients may be at higher risk of needing a tracheostomy was limited during the initial COVID-19 outbreak due to the novelty of the virus and unique infection control challenges of the procedure. International guidelines evolved quickly. Both the American Academy of Otolaryngology–Head and Neck Surgery (AAO-HNS) and ENT UK recommended delaying tracheostomy for at least 14 days post-intubation until viral shedding decreased [10]. Later studies confirmed that while delayed tracheostomy reduced staff exposure, it often prolonged sedation, mechanical ventilation, and ICU stay, emphasizing the need for more individualized decision-making [3].

Reports suggested that tracheostomy rates among intubated COVID-19 patients varied widely, ranging from 10% to 46%, depending on institutional policy, patient severity, and pandemic phase [11,12,13]. Despite the extensive literature on timing and safety guidelines, the question of which patients would ultimately require tracheostomy remains unexplored.

In non-COVID ARDS populations, studies have shown that patients who are older and have a lower P/F ratio, underlying respiratory conditions, and various lab findings are more likely to require prolonged ventilatory assistance or undergo tracheostomy [14]; however, specific literature on risk factors in a specific population with COVID-19 infection are scarce.

The aim of this study is to determine whether there are risk factors that can predict a patient’s need for tracheostomy, looking at patients who required intubation due to COVID-19 infection between 1 March 2020 and 31 December 2020.

## 2. Materials and Methods

### 2.1. Study Design and Population

This was a single-center retrospective cohort study of all adult patients intubated for COVID-19-related respiratory failure at Beth Israel Deaconess Medical Center (Boston, MA, USA) between 1 March 2020 and 31 December 2020. Patients were excluded if they tested negative for SARS-CoV-2 or were intubated for non-COVID-19 indications.

### 2.2. Inclusion and Exclusion Criteria

Inclusion Criteria:

- Adult patients (≥18 years);
- Laboratory-confirmed SARS-CoV-2 infection;
- Endotracheal intubation for COVID-19-related respiratory failure;
- Intubation between 1 March and 31 December 2020.

Exclusion Criteria:

- Intubation for non-COVID-19 indications;
- Negative SARS-CoV-2 testing;
- Death prior to tracheostomy;

Incomplete data for key variables.

### 2.3. Data Collection

Demographic data, including patient age, sex, smoking status, past medical history, and BMI, were collected, alongside information pertaining to the patient’s hospital course, hospital transfers, and complications. History of arrhythmia was determined based on documented diagnoses in the electronic medical record prior to admission, with atrial fibrillation being the most common arrhythmia identified. Details from the patient’s ICU stay pertaining to their course of mechanical ventilation and tracheostomy and its associated complications were also recorded. Patients who passed away while intubated and prior to tracheostomy were excluded from the analysis (*n* = 2). Immune-compromised status was determined by the documented use of immunosuppressive medications or documented immune-compromised status in the electronic medical record notes. Specific laboratory results, including white blood cell count, platelets, bilirubin, and creatinine, were collected from the day of intubation. Serum creatinine values were recorded in mg/dL from laboratory testing obtained on the day of intubation or within 24 h if unavailable. When results from the day of intubation were not available, results from the subsequent 24 h were considered. Oxygenation was assessed using the PaO_2_/FiO_2_ (P/F) ratio, calculated using arterial blood gas values and ventilator settings. The lowest P/F ratio within the first 48 h following intubation was recorded for analysis.

### 2.4. Study Objectives

The primary outcome of this study was to determine the risk factors for requiring tracheostomy among intubated patients with COVID-19 between 1 March 2020 and 31 December 2020. Secondary outcomes included a description and comparison of baseline characteristics of the study cohort.

### 2.5. Statistical Analysis

Continuous variables were represented as means and standard deviation and categorical variables were represented by frequency and proportion. Continuous variables were assessed for normality using the Shapiro–Wilk test. As none of the variables exhibited normality, a comparison of continuous variables between the tracheostomized and non-tracheostomized patients was performed using the Wilcoxon test. Categorical variables between the groups were compared using the Chi-squared tests and Fisher’s exact test. Univariate analysis was performed with the generalized linear model function. Statistical significance was set at *p* < 0.05. A biostatistician was consulted about the analysis plan. Statistical analyses were performed using R software (version 4.3.1; R Foundation for Statistical Computing, Vienna, Austria). 

Given the limited number of tracheostomy events, multivariable regression analysis was not performed to avoid model overfitting. All associations should therefore be interpreted as exploratory and hypothesis-generating. Patients with missing data for key variables were excluded from the analysis; no data imputation was performed.

### 2.6. Ethics

The study protocol was approved by the Institutional Review Board (IRB) of Beth Israel Deaconess Medical Center. The requirement for informed consent was waived due to the retrospective nature of the study and use of de-identified data.

## 3. Results

**Figure 1:**
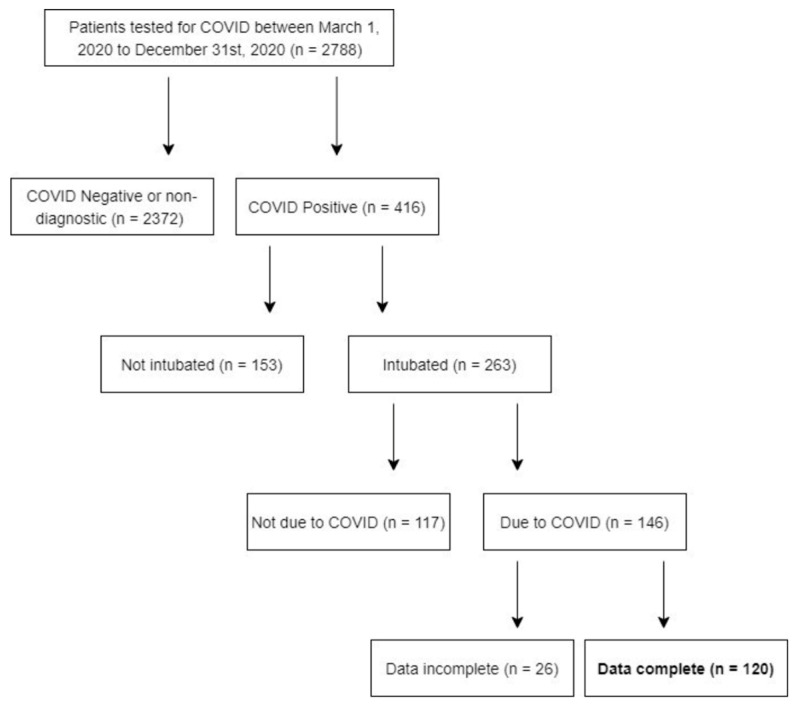
Flow diagram of patients tested for COVID-19 between 1 March and 31 December 2020 (n = 2788). Of these, 416 tested positive, with 263 requiring intubation. Among intubated patients, 146 were intubated due to COVID-19, of whom 120 had complete data for analysis.

In total, 120 patients intubated due to COVID-19 infection were included in our retrospective review based on the inclusion and exclusion criteria (Figure 1). Patients who died after intubation and prior to tracheostomy were also excluded from the analysis. Of the 120 patients included in the final analysis, 94 were extubated directly, and 26 underwent tracheostomy. In patients who did not eventually undergo a tracheostomy, the mean number of days from admission to intubation was 2.17 with a standard deviation of 2.97. Alternatively, in those that underwent tracheostomy, the mean number of days from admission to intubation was 1.81 with a standard deviation of 2.15. There was no statistically significant difference in the mean number of days from admission to intubation between these two groups (*p* = 0.898).

Patients who underwent tracheostomy were significantly more likely to be older than those who did not undergo tracheostomy (*p* = 0.009). Patients who underwent tracheostomy were also more likely to have higher creatinine values (*p* = 0.013), have lower PF ratios (*p* = 0.034), and have a history of arrhythmia (*p* = 0.012) compared to patients who did not undergo tracheostomy (Table 1).

Univariate analysis of the variables similarly revealed that age (OR 1.04, 95% CI: 1.01–1.09, *p* = 0.014), PF ratio (OR 0.991, 95% CI: 0.982–0.999, *p* = 0.034), and history of arrythmia (OR = 4.58, 95% CI: 1.42–14.93, *p* = 0.010) were independently associated with the need for tracheostomy. Creatinine values were not shown to be independently associated with tracheostomy in this univariate analysis (OR = 1.30, 95% CI: 0.99–1.77, *p* = 0.063) (Table 2).

## 4. Discussion

In this single-center retrospective cohort of 120 intubated COVID-19 patients, the results showed that older age, a lower PaO_2_/FiO_2_ (P/F) ratio, and a history of arrhythmia were significantly associated with subsequent need for tracheostomy. Laboratory values such as serum creatinine on the day of intubation also demonstrate some correlation with the need for tracheostomy, although this was not seen in the univariate analysis. These findings add to the literature by identifying early markers at intubation or shortly thereafter that may anticipate which patients will move to tracheostomy.

Most of the published literature has mainly addressed the timing of tracheostomy rather than baseline predictors of needing it. A meta-analysis of 14 studies including 2371 tracheostomized COVID-19 patients documented that early tracheostomy was correlated with shorter invasive mechanical ventilation duration (mean difference ~9 days) and shorter intensive care unit stay (mean difference ~9 days), without a difference in mortality (OR 1.09, 95% CI 0.79–1.51) [15]. On a similar note, Grotowska et al. reported that early tracheostomy reduced ventilator-associated pneumonia, duration of ventilation (18 vs. 33 days), and ICU/hospital LOS [16]. These studies note that timing matters, but do not indicate which patients should be selected earlier.

Sancho et al. identified the SOFA score at ICU admission as the lone independent predictor of tracheostomy need (cut-off 4.5) [17]. In our cohort, the P/F ratio (a marker of gas-exchange impairment) and arrhythmia history (perhaps reflecting cardiac reserve or systemic illness severity) may serve as analogous early indicators. Older age, likewise, has been widely shown to worsen COVID-19 severity and predicts the need for prolonged ventilation and ICU resources, aligning with our results.

Old age may be associated with reduced respiratory muscle reserve, diminished capacity for recovery from ARDS, and greater comorbidity burden [14,18]. Moreover, a lower P/F ratio shortly after intubation suggests more severe alveolar injury, reduced lung compliance or ongoing high ventilatory support, which are all factors that may delay extubation and favor tracheostomy. In this study, the P/F ratio was used to assess oxygenation due to its feasibility and practicality based on the existing available data. We recorded the lowest P/F ratio observed in the first 48 h after intubation. Patients with lower P/F ratios immediately following intubation were more likely to need a tracheostomy than those with higher ratios, which is in line with what was expected to be observed in this population.

Among patients that had a history of arrhythmia, most had atrial fibrillation. Previous studies assessing the relationship between atrial fibrillation and COVID-19 identified that AF (both newly diagnosed and pre-existing) is associated with increased rates of morbidity and mortality following infection with COVID-19 [19,20,21].

COVID-19 is a uniquely challenging condition due to the prevalence of ARDS [2], particularly during the initial wave, which made identifying risk factors particularly important in appropriately triaging need while working with limited medical resources. Additionally, because of the high risks to healthcare workers during the initial wave, tracheostomy was often delayed due to the documented concerns with aerosol-generating procedures during this time [22]. In patients with mild or moderate COVID-19, patients are usually not infectious after 10 days; however, this period can be much longer in those who are critically unwell [23]. Most guidelines surrounding tracheostomies suggest that we should delay the procedure to 14 days and only consider it in patients with stable pulmonary status [24]. Identification of these risk factors at or soon after intubation may allow clinicians to anticipate the likelihood of tracheostomy, enabling earlier multidisciplinary planning (surgeon, intensivist, respiratory therapy), family counselling, and resource allocation.

### 4.1. Study Limitations

There are several limitations to this study that must be considered when interpreting the results. Firstly, our study focused on a specific respiratory condition, COVID-19, and primarily looked at the first and second waves of the virus. Nonetheless, COVID-19 remains a concern, with recent increases in patients with active infection seeking medical attention. Studies on SARS in 2003 were also important in helping us navigate the treatment of COVID-19 [25], and findings from our examination of COVID-19 will undoubtedly be meaningful in future pandemics. Clinical practice during the early phases of the COVID-19 pandemic varied considerably, including criteria for intubation, ventilator management, sedation strategies, and timing of the tracheostomy. Concerns related to personal protective equipment availability and aerosol-generating procedures may have influenced clinical decision-making. These temporal and institutional factors may limit the applicability of the findings to later phases of the pandemic or to contemporary practice. This study was also focused on a single treatment center and had a small sample size, which limits the generalizability of the study. Due to the small number of patients in the tracheostomy group, there were not enough patients to build a reliable predictive model using multivariate regression. Because the analysis was limited to univariate associations, the independence of the identified predictors cannot be established. Residual confounding related to illness severity, ventilatory strategies, sedation practices, and extubation attempts may have influenced the observed associations. Finally, there was no comparison group for this observational study.

### 4.2. Future Perspectives

Looking forward, future studies should aim to validate these findings in larger, multicenter cohorts and assess independent predictors using multivariable modeling. The data can be assessed with the above findings to formulate a validated score that can predict respiratory outcomes in COVID-19 and for future respiratory viruses. As COVID-19 cases surge and mutations continue to develop, we can apply findings from earlier phases of the virus outbreak to address current challenges in the care of our patients.

## 5. Conclusions

In this cohort of COVID-19 patients intubated for respiratory failure, older age, lower P/F ratio, and a history of arrhythmia were identified as early predictors of tracheostomy. These findings highlight the importance of early risk stratification to guide tracheostomy planning and optimize critical-care resource allocation. Such insights may help clinicians anticipate care needs earlier in the course of mechanical ventilation.

## Figures and Tables

**Table 1:** Comparison of variables between tracheostomized and non-tracheostomized patients (* *p* < 0.05).

	No Tracheostomy	Tracheostomy	*p*
Sample size (*n*)	94	26	--
Demographic variables			
Age, mean (SD)	57.93 (14.43)	65.85 (11.43)	0.009 *
Female, n (%)	40 (42.6)	11 (42.3)	0.982
BMI, mean (SD)	32.16 (7.70)	32.82 (7.36)	0.624
Transferred from other hospital, n (%)	43 (45.7)	16 (61.5)	0.154
Non-smoker, n (%)	53 (56.4)	15 (57.7)	0.905
Lab values (mean, SD)			
WBC, K/uL	8.54 (4.34)	9.09 (3.75)	0.328
Platelets, k/UL	239.03 (79.78)	237.50 (84.50)	0.816
Bilirubin, mg/dL	0.51 (0.35)	0.53 (0.36)	0.524
Creatinine, mg/dL	1.24 (1.18)	1.88 (1.19)	0.013 *
P/F ratio, mmHg	149.97 (67.11)	119.31 (44.21)	0.034 *
Co-morbidities, n (%)			
Lung disease	21 (22.3)	10 (38.5)	0.096
*COPD*	3 (3.2)	3 (11.5)	0.115
*OSA*	9 (9.6)	4 (15.4)	0.475
*Asthma*	6 (6.4)	4 (15.4)	0.221
*Other lung condition*	5 (5.3)	0 (0.0)	0.584
Heart disease	16 (17.0)	7 (26.9)	0.256
*Arrhythmia*	7 (7.4)	7 (26.9)	0.012 *
*Heart failure*	5 (5.3)	3 (11.5)	0.368
*Coronary artery disease*	7 (7.4)	2 (7.7)	1.000
*Other heart condition*	4 (4.3)	1 (3.8)	1.000
Chronic kidney disease	9 (9.6)	4 (15.4)	0.476
Diabetes mellitus	37 (39.4)	11 (42.3)	0.786
Cancer	3 (3.2)	0 (0.0)	1.000
Immune-compromised status	4 (4.3)	4 (15.4)	0.066

**Table 2:** Univariate comparison of tracheostomized and non-tracheostomized patients (* *p* < 0.05).

	OR	95% CI	*p*
Age	1.04	1.01–1.09	0.014 *
Female	0.99	0.40–2.37	0.982
BMI	1.01	0.95–1.07	0.695
Transfer	1.90	0.79–4.74	0.157
Non-smoker	0.95	0.39–2.27	0.905
WBC	1.03	0.93–1.14	0.552
Platelets	1.00	0.99–1.01	0.931
Bilirubin	1.80	0.74–4.54	0.176
Creatinine	1.30	0.99–1.77	0.063
P/F ratio	0.991	0.982–0.999	0.034 *
COPD	3.96	0.69–22.63	0.105
OSA	1.72	0.43–5.83	0.403
Asthma	2.67	0.64–10.17	0.154
Arrhythmia	4.58	1.42–14.93	0.010 *
Heart Failure	2.32	0.45–10.19	0.272
Coronary artery disease	1.04	0.15–4.63	0.966
Chronic kidney disease	1.72	0.43–5.83	0.403
Diabetes mellitus	1.13	0.46–2.72	0.786
Immune-compromised status	4.09	0.90–18.57	0.059

## Data Availability

Data is available upon request.

## Abbreviations

The following abbreviations are used in this manuscript:

COVID-19	Coronavirus disease 2019
P/F ratio	Ratio of arterial oxygen partial pressure (PaO_2_) to the fraction of inspired oxygen concentration (FiO_2_)
ICU	Intensive care unit
PaO_2_	Arterial oxygen partial pressure
OR	Odds ratio
ARDS	Acute respiratory distress syndrome
SARS	Severe acute respiratory syndrome
